# Genomic Analyses of Methicillin-Susceptible and Methicillin-Resistant *Staphylococcus pseudintermedius* Strains Involved in Canine Infections: A Comprehensive Genotypic Characterization

**DOI:** 10.3390/pathogens13090760

**Published:** 2024-09-04

**Authors:** Maria Eduarda Rocha Jacques da Silva, Gabriela Merker Breyer, Mateus Matiuzzi da Costa, Bertram Brenig, Vasco Ariston de Carvalho Azevedo, Marisa Ribeiro de Itapema Cardoso, Franciele Maboni Siqueira

**Affiliations:** 1 Department of Veterinary Clinical Pathology, Faculty of Veterinary Medicine, Federal University of Rio Grande do Sul, Porto Alegre 91540-000, Brazil; mariaeduardaroocha@hotmail.com (M.E.R.J.d.S.); gabibreyer@hotmail.com (G.M.B.); 2 Postgraduate Program in Veterinary Science, Federal University of Rio Grande do Sul, Porto Alegre 91540-000, Brazil; 3 Department of Animal Science, Federal University of São Francisco Valley (UNIVASF), Petrolina 56300-000, Brazil; 4 Institute of Veterinary Medicine, Division of Molecular Biology of Livestock and Molecular Diagnostics, Georg August University Göttingen, 37077 Göttingen, Germany; 5 Molecular and Cellular Genetics Laboratory (LGCM), Institute of Biological Sciences, Federal University of Minas Gerais (UFMG), Belo Horizonte 31270-901, Brazil

**Keywords:** resistance, virulence, zoonosis, MSSP, MRSP, wgNGS

## Abstract

*Staphylococcus pseudintermedius* is frequently associated with several bacterial infections in dogs, highlighting a One Health concern due to the zoonotic potential. Given the clinical significance of this pathogen, we performed comprehensive genomic analyses of 28 *S. pseudintermedius* strains isolated from canine infections throughout whole-genome sequencing using Illumina HiSeq, and compared the genetic features between *S. pseudintermedius* methicillin-resistant (MRSP) and methicillin-susceptible (MSSP) strains. Our analyses determined that MRSP genomes are larger than MSSP strains, with significant changes in antimicrobial resistance genes and virulent markers, suggesting differences in the pathogenicity of MRSP and MSSP strains. In addition, the pangenome analysis of *S. pseudintermedius* from canine and human origins identified core and accessory genomes with 1847 and 3037 genes, respectively, which indicates that most of the *S. pseudintermedius* genome is highly variable. Furthermore, phylogenomic analysis clearly separated MRSP from MSSP strains, despite their infection sites, showing phylogenetic differences according to methicillin susceptibility. Altogether our findings underscore the importance of studying the evolutionary dynamics of *S. pseudintermedius*, which is crucial for the development of effective prevention and control strategies of resistant *S. pseudintermedius* infections.

## 1. Introduction

*Staphylococcus pseudintermedius* is a commensal bacteria frequently found in the mucosal tissue and skin of mammals [1]. In dogs, some virulent *S. pseudintermedius* strains cause opportunistic infections [2], including pyoderma, otitis, cystitis, pyometra, bacteremia and post-surgical infections [1,3,4]. The effective treatment of such infections may be hampered by the high virulence of *S. pseudintermedius* and the emergence of antimicrobial resistance among the circulating clinical specimens [5,6].

Accordingly, *S. pseudintermedius* with clinical importance are classified as methicillin-susceptible *S. pseudintermedius* (MSSP) or methicillin-resistant *S. pseudintermedius* (MRSP), based on the presence of *mecA* gene. This gene encodes a penicillin-binding protein 2a (PBP2a), which confers resistance to beta-lactam antibiotics, including methicillin [7]. Beta-lactams are a crucial class of medications frequently used to treat bacterial infections in canines. Although multidrug resistance (MDR) is commonly observed in MRSP, some MSSP also can show a MDR profile [5,8]. Therefore, understanding the differences between MSSP and MRSP is essential for both monitoring their role in the spread of antimicrobial resistance patterns and their impact on animal health.

Furthermore, *S. pseudintermedius* is also a concern for One Health due to its zoonotic potential; thus, it is necessary that more studies study this issue, regarding the characterization of the pathogenicity profile of canine clinical strains and their dissemination potential to humans. Hence, comparative genomic analyses based on next generation sequencing (NGS) emerge as an impressive tool to enhance the comprehension about the epidemiology, antimicrobial susceptibility, pathogenicity, genetic diversity and evolutive dynamics of *S. pseudintermedius* isolated from clinical infections [9,10,11].

Therefore, this study performed a comprehensive genomic characterization of 28 *S. pseudintermedius* strains isolated from canine infections, considering MRSP and MSSP strains. In addition, we performed a comparative genomic analysis with *S. pseudintermedius* from human infections to assess the relation of canine and human isolates and infer the zoonotic potential of the investigated *S. pseudintermedius* canine strains.

## 2. Materials and Methods

### 2.1. Bacterial Strains and DNA Isolation

For this study, 28 *S. pseudintermedius* strains previously isolated from canine infections between 2017 and 2018 were investigated, including cases of otitis (n = 5), pyodermatitis (n = 14), pyometra (n = 5), cystitis (n = 2) and sepsis (n = 2). The strains were classified as MRSP (n = 15) or MSSP (n = 13) based on the *mecA* gene presence [5]. The clonal diversity of the strains was previously assessed using multilocus sequence typing (MLST) [5]. The total DNA was isolated directly from colonies that underwent 24 h culture at 37 °C in Tryptic Soy Agar (TSA; Difco, Franklin Lakes, NJ, USA). For cell lysis, the colonies were suspended in 200 μL of lysis-buffer (25 mM Tris–HCl pH 8, 2,5 mM EDTA, 1% TRITON-100X) containing lysozyme (20 mg/mL) and lysostaphin (1 mg/mL), incubated at 37 °C for 18 h. Then, DNA extraction was performed using a Purelink Genomic DNA Kit (Thermo Fisher Scientifics, MA, USA), according to the manufacturer’s instructions. DNA samples were quantified using Qubit*^®^* (Life technologies, Grand Island, NY, USA) and DNA quality was assessed using NanoDrop Lite (Thermo Fischer Scientific, Waltham, MA, USA).

### 2.2. Whole-Genome Sequencing and Analysis of Canine Staphylococcus pseudintermedius Strains

DNA libraries were prepared and submitted for 2 × 250 bp Illumina HiSeq sequencing (HiSeq*^®^* Reagent Kit v2, 500 cycles), according to the manufacturers’ instructions (Illumina, San Diego, CA, USA). The raw reads quality was assessed using FastQC 0.11.9 [12], then adapter, short-length (>150 nt) and low-quality (Phred ≥ 30) sequences were trimmed using Trimmomatic 0.39 [13]. Genome assembly was performed using Edena 3 [14], while plasmid sequences identification and segregation was made using two programs: MOB-suite 3.0.3 [15] and PlasmidFinder 2.1 [16]. The IMAGE 2.4.1 [17] was used to close sequence gaps after assembly. Genome quality was assessed using Quast 5.2 [18]; then, the genomes were aligned against the referential *S. pseudintermedius* SP_113043A (NZ_CP065921) using Mauve 2.4.0 [19]. Afterward, genome annotation was performed using Prokka 1.14.5. [20].

### 2.3. Resistance and Virulence Genotypic Characterization

The 28 *S. pseudintermedius* genomes were submitted to an in silico prediction of several genetic elements. Briefly, CRISPRCasFinder [21], PHASTER [22], IslandViewer 4 [23], Isfinder [24], antiSMASH 7.0 [25] and SCC*mec*Finder 1.2 [26] were used to identify Clustered Regularly Interspaced Short Palindromic Repeats (CRISPR), prophages, genomic islands (GI), insertion sequences (IS), biosynthetic gene clusters (BGC) and elements of the staphylococcal cassette chromosome *mec* (SCC*mec*), respectively.

In addition, antimicrobial resistance genes (ARG) were predicted through the compiling of several research databases: ResFinder 4.1 [27], Resistance Gene Identifier (RGI) [28] with the Comprehensive Antibiotic Resistance Database (CARD) [28], ABRIcate 1.0.0 [29] and Patric [30] in conjunction with the National Database of Antibiotic Resistant Organisms (NDARO) [31]. Alternatively, virulence genes were predicted using the Virulence Factor Database (VFDB) [32] and Patric using the Victors Database [33]. For the data, we only considered predictions with ≥80% coverage, ≥80% identity and an E-value <10^−5^.

### 2.4. Comparative Genomics and Phylogenomic of Staphylococcus pseudintermedius from Canine and Human

To assess the phylogenetic relation and zoonotic potential of the investigated canine *S. pseudintermedius* strains, we retrieved other *S. pseudintermedius* strains from the National Center for Biotechnology Information (NCBI): two from dogs (NZ_CP065921 and CP066702.1) and four from humans (NZ_CP045086.1, NZ_CP031561.1, NZ_CP031605.1, and NZ_CP030715.1) (Table S1). Pangenome analysis was performed using Roary 3.11.2 [34] to assess the core and accessory genome from the analyzed dataset (n = 34); Phandango [35] was used for visualization. Then, a phylogenomic tree of the *S. pseudintermedius* core genome was built with Maximum Likelihood (ML) using the General Time Reversible (GTR; bootstrap = 1000) in the MEGA11 [36].

### 2.5. Statistical Analyses

The genome length of the MRSP and MSSP strains was compared via Wilcoxon test (*p* < 0.05) [37,38]. Data visualization and analysis were performed using R 4.3.1. [39]. For comparing the number of islands between MRSP and MSSP groups, a t-test was conducted using WinPEPI 11.65 [40].

## 3. Results

### 3.1. Genome Characterization of Staphylococcus pseudintermedius from Canine Infections

The comparative genome analysis demonstrated that the genome length of the investigated canine *S. pseudintermedius* strains ranged from 2.4 to 2.8 Mb (Table 1), being longer in MRSP than in MSSP (*p* < 0.05) (Figure S1). The mapping statistics of genomes sequencing and assembly showed a 92 to 236X coverage, the contigs count ranged from 34 to 182, N50 from 29,816 to 190,599 bp, and G + C content from 37.3 to 37.7%. The number of predicted coding sequences (CDS) ranged from 2342 to 2868.

Considering the prediction of genetic elements in the *S. pseudintermedius* strains, we observed a high number of GI, ranging from 3 to 19 (Table 2), with an average of 10.2 GI in MSSP and 16.2 GI in MRSP strains, with the number being higher in MRSP (*p* < 0.05). Moreover, the discovered number of prophages per genome in the analyzed strains was low, with similar counts between MRSP and MSSP (Table 2).

The SCC*mec* typing showed that the SCC*mec* type III was the most common among the investigated strains, whereas only the *S. pseudintermedius* 1044, a MRSP strain, belonged to type V (Table 2). In total, 18% (5/28) of the *S. pseudintermedius* strains showed a complete CRISPR/Cas system, with four being MSSP and one being MRSP (Table 2). The prediction of BGC ranged from 5 to 11 among the investigated genomes (Table 2), being identified clusters related to non-ribosomal peptide synthetases (NRPS), ribosomally synthesized and post-translationally modified peptides (RiPPs), linear azol(in)e-containing peptides (LAPs) and an opine-like metallophore. In addition, we observed a highly variable number of IS: 52 were predicted in MRSP strains, with 5 being exclusives from this group (IS1272, ISLac1, ISSau3, ISSau5, and ISSep1); 81 IS were predicted for the MSSP strains, with 34 being exclusives for this MSSP group, such as ISSag12, ISS1X, ISLmo14, and IS1469 (Table S2).

The plasmid search using PlasmidFinder and MOB-suite identified plasmids in 6 MSSP strains and 13 MRSP strains, all of which have been previously described in other *Staphylococcus* spp. (Table S3). In detail, in both MSSP and MRSP groups, we identified the plasmid p222, which harbors the *bcrA* gene and whose product confers resistance to bacitracin (Table S3).

### 3.2. Pathogenicity Profile of the Staphylococcus pseudintermedius Strains from Canine Infections

**Figure 1:**
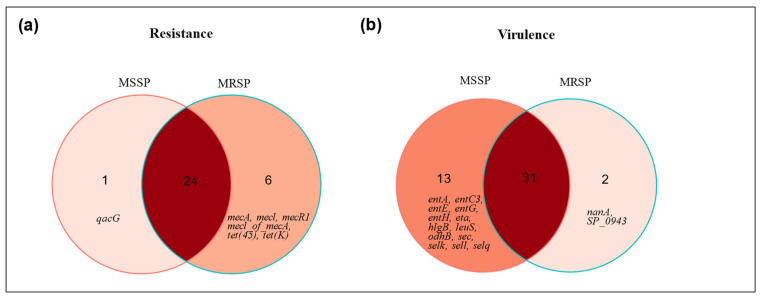
Venn diagram of antimicrobial resistance genes and virulence markers in MRSP and MSSP strains isolated from canine. (**a**) Comparison of antimicrobial resistance genes between MSSP and MRSP; (**b**) comparison of virulence genes between MSSP and MRSP.

**Figure 2:**
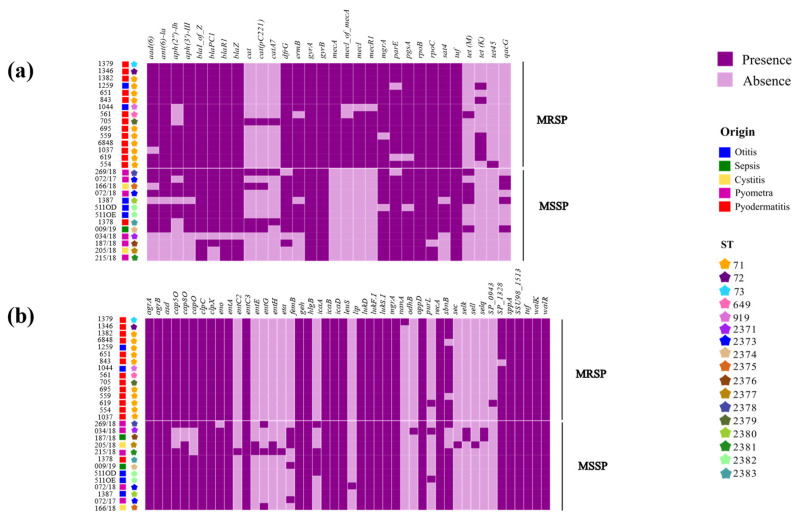
Heatmap representation indicating the presence (in purple) and absence (in lilac) of virulence genes and antimicrobial resistance marker genes across all 28 genomes of *Staphylococcus pseudintermedius*. (**a**) The distribution of antimicrobial resistance marker genes; (**b**) the distribution of virulence genes.

The in silico prediction of ARG suggested that all *S. pseudintermedius* strains were multidrug resistant, as 100% of the genomes harbored genes from at least three antimicrobial drug classes (Table S4). We observed that MRSP have more genes involved in antimicrobial resistance than MSSP (Figure 1a; Table S4). A total of 24 ARGs were shared between MRSP and MSSP, and both groups have exclusive ARGs. In detail, only one exclusive ARG was predicted in MSSP (*qacG*), whereas MRSP harbored six exclusive ARGs (*mecA*, *mecl*, *mecR1*, *mecI of mecA*, *tet(45)*, and *tet(K)*). Accordingly, the general ARGs distribution indicates differences between the MSSP and MRSP groups (Figure 2a), with a more consistent and homogeneous ARG profile in the MRSP strains, whereas the MSSP group exhibits greater ARG diversity.

On the other hand, comparing the number of virulence genes in the investigated *S. pseudintermedius* strains, we determined a higher number of virulence genes in MSSP than in MRSP (Figure 1b; Table S4). A total of 31 virulence genes were shared between the analyzed groups; 13 genes were found only in MSSP strains (*entA*, *entC3*, *entE*, *entG*, *entH*, *sec*, *sell*, *selq*, *selk*, *eta*, *hlgB*, *leuS*, and *odhB*), and only 2 exclusive predicted virulence factors were found in the MRSP strains (*SP_0943* and *nanA*). In addition, distinct virulence profiles were observed when comparing MSSP and MRSP groups; despite the fact that most virulence genes are shared between MRSP and MSSP strains, MSSP strains showed a more diverse profile (Figure 2b).

### 3.3. Comparative Genomics and Phylogenomic of Staphylococcus pseudintermedius from Canine and Human Hosts

The genomes of *S. pseudintermedius* from multiples sources and hosts were analyzed, including the 28 from canine infections from this study, and 6 were retrieved from NCBI, representing isolates from human and canine origins (Table S1). In detail, canine MRSP strains were associated with otitis and pyoderma cases, whereas MSSP strains were isolated from cases of pyometra, sepsis, cystitis and otitis; among the *S. pseudintermedius* strains from humans, there were four MRSP strains previously isolated from skin samples and mixed cultures. The MLST analyses showed that sequence types (STs) varied considerably among the analyzed strains, regardless of their origin. ST71 was the most frequent type, circulating predominantly among the canine hosts in the analyzed genomes (Table S1).

**Figure 3:**
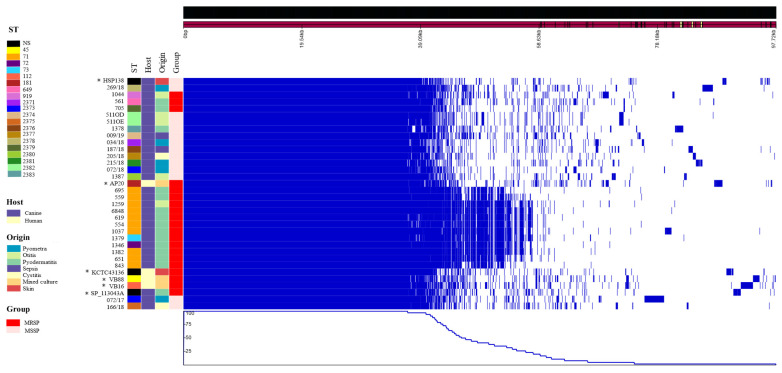
Genomic analysis of 34 isolates of *Staphylococcus pseudintermedius* related to presence and absence of genes by homology. ST: Sequencing Type based on MLST. NS: Not specified. “*” = Genomes retrieved from NCBI database.

We identified a total of 4884 genes in the analyzed *S. pseudintermedius* genomes, with 1847 genes as part of the core genome and 3037 belonging to the accessory genome. The overall genomic analysis highlighted the conserved and non-conserved regions among the investigated genomes (Figure 3). We observed that the ST71 of *S. pseudintermedius*, associated with pyoderma, exhibited a more conserved region compared to other strains, grouping together cohesively. Overall, there was generally a closer alignment among isolates according to the MSSP or MRSP groups, except for isolates 072/17, 166/18, 1044, 561 and 705 (Figure 3).

**Figure 4:**
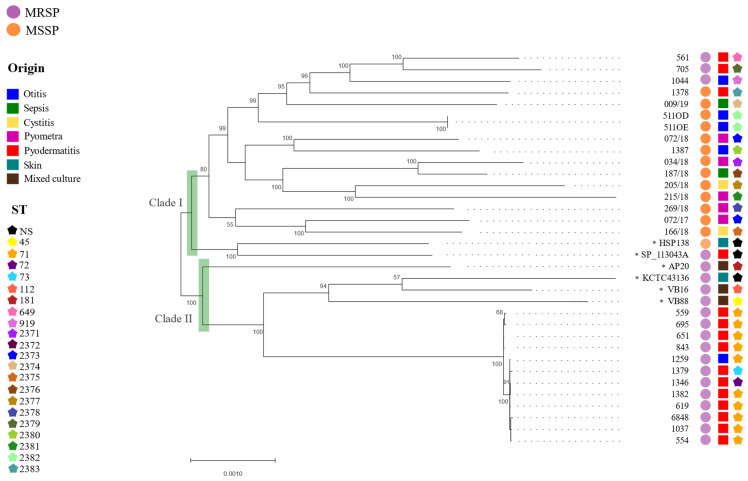
Phylogenomic analysis of 34 *Staphylococcus pseudintermedius* strains based on core genome. The phylogenetic tree was built using Maximum Likelihood method (bootstrap = 1000; General Time Reversible model) in MEGA11. ST: Sequencing Type based on MLST. “*” = Genomes obtained from NCBI database.

The phylogenomic analysis of *S. pseudintermedius* based on 1847 genes from the core genome grouped the analyzed genomes in two separate clades: (i) clade I clustered 18 *S. pseudintermedius*, mostly belonging to MSSP group except for four strains (561, 705, 1044 and SP_113043A), and (ii) clade II that exclusively comprised MRSP strains (Figure 4). Moreover, no phylogenetic clusterization was observed related to the isolation source. In detail, in clade I, the four MRSP strains were isolated from pyoderma cases and show diverse sequence types (ST649, ST919 and ST2379). In clade II, most of the *S. pseudintermedius* strains from pyoderma were identified as ST71, except the canine strains 1346 and 1379, which were classified as ST72 and ST73, respectively. Among the human strains in clade II, *S. pseudintermedius* AP20 (ST181) stands out by clearly separating from the others (Figure 4). Figure 4 shows that the selected human *S. pseudintermedius* were classified into different STs.

## 4. Discussion

The high occurrence of *S. pseudintermedius* infections with the MDR profile in dogs, associated with the zoonotic potential of some clinical strains, encourages studies regarding the genotypic characterization of MSSP and MRSP strains with epidemiological importance. Hence, in this study, we performed a comprehensive genomic characterization of *S. pseudintermedius* strains isolated from canine infections that provided insights into genetic variations and the potential zoonotic transmission of the pathogen, based on the pangenomic comparison of strains from human and canine origins.

The phenotypical characterization and genomic virulence potential of the canine *S. pseudintermedius* strains used in this study were described in a previous study [5]. Among the analyzed canine isolates, *S. pseudintermedius* ST71 was the most predominant, which is often associated with pyoderma and has a wide geographic distribution [41], being the most prevalent clone in Europe [42] and commonly found in Brazil [3,5,43]. Nevertheless, recent evidence suggests a decline in ST71 prevalence, accompanied by the emergence of ST258 [44]. In addition, our findings also identified *S. pseudintermedius* ST68, ST69 and ST90, reflecting a diversity of sequence types currently in circulation in dogs in Brazil.

Genome analysis determined that the genome size of the 28 *S. pseudintermedius* strains analyzed in this study are within the expected amount for this species [10,45,46]. Moreover, the MRSP genomes were significantly larger than the MSSP ones, which is in accordance with previous data [10]. From the search for genetic features, we highlight the prediction of IS1272 exclusively in the MRSP group in accordance with previous data that associated this IS to MRSP strains, usually located downstream of the *mecR* gene [47], suggesting that IS1272 may serve as a distinctive marker for the MRSP group. In MSSP, ISSag12 was exclusively found in these strains, being associated with the truncation of *sat4* that encodes streptomycin adenyltransferase related to streptomycin resistance [48].

In terms of pathogenicity, we predicted ARGs and virulence genes and compared pathogenicity patterns between MSSP and MRSP, which enable us to identify exclusive genetic elements involved in the pathogenesis of such strains. In MSSP strains, *qacG* was the only exclusive ARG, which confers resistance to disinfectants, whereas in MRSP, exclusive ARG included genes involved in methicillin resistance (*mecA, mecl* and *mecI of mecA*), as expected, and genes related to tetracycline resistance (*tet(45)* and *tet(K)*). In agreement with our findings, a previous study assessing the phenotypic antimicrobial susceptibility of MRSP strains isolated from dogs with otitis has also identified a high occurrence of MRSP strains resistant to tetracycline (61.4%) [49]. Even though MRSP strains are commonly resistant genes for several non-beta-lactam drugs, including tetracycline, this antimicrobial drug is still a choice for treating *S. pseudintermedius* infections in small animals [50]. Altogether, the presence of *tet(45)* and *tet(K)* in MRSP genomes might suggest that these isolates were subjected to more intense selective pressure, potentially due to the prolonged or excessive use of such antimicrobials.

Additionally, the in silico prediction of ARGs determined that all analyzed *S. pseudintermedius* strains harbored antimicrobial resistance genes associated with at least three different classes of antibiotics, suggesting a potential MDR profile; however, in the previous phenotypic analysis, not all strains exhibited an MDR in vitro profile, with 25 strains being classified as MDR in the phenotypic analysis [5].

Overall, we observed genotypic homogeneity among MRSP isolates when considering ARGs distribution, which suggests that MRSP isolates share a common set of resistance genes, potentially associated with specific mechanisms of antimicrobial resistance. In contrast, the MSSP group exhibits greater genotypic diversity, evidenced by the variable presence of antimicrobial resistance genes among the isolates, reflecting a more diverse adaptation to the environments in which they were found.

Considering exclusive virulence genes, MSSP harbored genes associated with enterotoxins production (*entA*, *entC3*, *entE*, *entG*, *entH*, *sell*, *selk*, *selq*), exfoliative toxins (*sec, eta)*, citotoxins (*leuS*), a gene related to metabolism (*odhB*), and a gene in immune system evasion (*hlgB*), whereas MRSP’s exclusive virulence genes encode N-acetylneuraminate lyase (SP_0943) and methyltransferase (*nanA*), which are involved in immune system evasion. In comparison, the main difference between MSSP and MRSP virulence profile lies in toxin genes. MSSP produces a wide variety of toxins, including enterotoxins and exfoliative toxins that are associated with tissue damage and infection dissemination [51]. In contrast, MRSP employs genes for immune evasion, such as N-acetylneuraminate lyase and methyltransferase, which facilitate its persistence in the host [51]. These differences in the range of exclusive virulence genes between MSSP and MRSP may reflect distinct strategies for pathogenicity and immune system evasion, highlighting the adaptive diversity of the two isolate groups.

The pangenome analysis of *S. pseudintermedius* revealed significant differences between isolates of human and canine origin, highlighting the complexity of zoonotic interactions. The core genome phylogenomic analysis revealed that human isolates clustered together with canine MRSP strains, which is particularly concerning from a One Health perspective, as it suggests a close phylogenetic relationship and a similar genome content among these strains that might facilitate interspecies transmission [52,53]. Moreover, to assess the zoonotic potential, the epidemiological distribution of *S. pseudintermedius* must be taken into account. As already mentioned, *S. pseudintermedius* ST71, which has a worldwide distribution and great epidemiological importance [43,54], was reported in both humans and dogs hosts analyzed in this study. Altogether, these findings highlight the ability of *S. pseudintermedius* clones to adapt to different hosts; therefore, there is a need for ongoing monitoring and control measures to address the zoonotic transmission and spread of resistant bacterial strains.

## 5. Conclusions

Overall, the genomic characterization of *S. pseudintermedius* strains isolated from infections in dogs revealed significant differences between MRSP and MSSP strains in terms of genetic characteristics, including the identification of potential antimicrobial resistance and virulence markers that improve the understanding of the pathogenicity of *S. pseudintermedius*. In addition, the comparison with strains from human infections provides insights into genetic variations and the possible zoonotic transmission of the pathogen. Our results emphasize the need for further studies on the zoonotic potential of *S. pseudintermedius* from canine infections and its ability to spread to humans in order to understand the evolutionary dynamics of this pathogen and to prevent the spread of resistant strains involved in canine infections.

## Figures and Tables

**Table 1:** The mapping statistics of the *Staphylococcus pseudintermedius* strains isolated from canine infections.

Identification	Contigs	N50	Total Size (bp)	Coverage (X)	GC (%)	CDS	tRNAs	rRNAs
166/18	54	154,498	2,629,757	151	37.45	2502	59	11
205/18	34	190,599	2,488,793	194	37.64	2342	59	14
072/17	65	131,476	2,709,226	230	37.31	2626	59	11
072/18	113	49,697	2,531,306	199	37.66	2371	59	11
215/18	49	122,689	2,535,717	236	37.48	2391	59	12
269/18	65	102,455	2,712,159	215	37.34	2626	59	9
034/18	87	60,695	2,492,124	193	37.70	2367	59	9
009/19	58	93,282	2,595,405	180	37.57	2509	59	10
187/18	40	138,227	2,547,925	227	37.53	2452	59	11
511OD	53	108,93	2,576,436	163	37.54	2472	59	13
511OE	79	74,309	2,580,431	226	37.54	2471	59	14
1044	79	57,906	2,623,021	228	37.44	2510	59	14
1259	82	83,218	2,846,852	153	37.33	2830	59	9
1387	39	172,802	2,567,762	167	37.48	2424	59	10
554	182	29,816	2,850,261	150	37.34	2804	59	9
559	58	107,552	2,572,158	166	37.40	2644	59	8
561	47	165,974	2,605,024	158	37.45	2495	59	10
619	155	37,758	2,848,699	118	37.33	2808	60	9
651	66	109,829	2,766,829	138	37.37	2716	59	9
695	100	120,157	2,686,739	128	37.40	2588	59	9
705	56	87,508	2,526,988	151	37.50	2391	59	10
843	69	89,873	2,688,725	155	37.41	2587	59	9
1037	78	75,758	2,881,635	152	37.30	2868	59	9
1346	89	102,677	2,820,424	142	37.35	2783	59	9
1378	66	107,552	2,575,532	92	37.43	2443	59	7
1379	114	65,855	2,851,013	140	37.33	2812	59	9
1382	65	133,050	2,818,793	147	37.35	2793	59	10
6848	81	88,449	2,844,513	148	37.33	2820	59	9

**Table 2:** In silico prediction of genetic elements in *Staphylococcus pseudintermedius* from canine infection.

Identification	MLST	Group	SCC*mec*	CRISPR	IS	GI	BGC	Prophage
166/18	ST2375	MSSP	-	Cas cluster, CRISPR	54	12	9	1
205/18	ST2377	MSSP	-	Cas cluster, CRISPR	18	6	7	0
072/17	ST2373	MSSP	-	CRISPR	22	14	8	0
072/18	ST2373	MSSP	-	-	38	7	5	1
215/18	ST2381	MSSP	-	-	28	12	6	0
269/18	ST2378	MSSP	-	CRISPR	48	14	10	3
034/18	ST2371	MSSP	-	Cas cluster, CRISPR	16	3	8	0
009/19	ST2374	MSSP	-	-	47	13	8	1
187/18	ST2376	MSSP	-	Cas cluster, CRISPR	17	9	9	0
511OD	ST2382	MSSP	-	CRISPR	28	10	8	2
511OE	ST2382	MSSP	-	CRISPR	28	10	8	1
1044	ST919	MRSP	V	Cas cluster, CRISPR	34	11	8	1
1259	ST71	MRSP	III	CRISPR	36	18	9	3
1387	ST2380	MSSP	-	CRISPR	15	9	9	0
554	ST71	MRSP	III	Cas cluster, CRISPR	36	17	9	2
559	ST71	MRSP	III	-	35	18	9	3
561	ST649	MRSP	-	CRISPR	43	12	7	2
619	ST71	MRSP	III	CRISPR	36	18	11	2
651	ST71	MRSP	III	-	36	17	9	3
695	ST71	MRSP	III	-	36	17	9	1
705	ST2379	MRSP	-	CRISPR	36	13	8	0
843	ST71	MRSP	III	CRISPR	36	19	9	1
1037	ST71	MRSP	III	CRISPR	36	16	9	3
1346	ST72	MRSP	III	CRISPR	36	17	9	1
1378	ST2383	MSSP	-	-	36	14	7	1
1379	ST73	MRSP	III	CRISPR	33	14	8	2
1382	ST71	MRSP	III	CRISPR	36	18	9	2
6848	ST71	MRSP	III	CRISPR	36	18	9	3

IS: Insertion Sequences; GI: Genomic Islands; BGC: Biosynthetic Gene Clusters; “-” indicates absence.

## Data Availability

The data underlying this article are available in the National Center for Biotechnology Information under BioProject PRJNA918652 and can be accessed with Accession Numbers JAQIAZ000000000, JAQIAY000000000, JAQIAX000000000, JAQIAW000000000, JAQIAV000000000, JAQIAU000000000, JAQIAT000000000, JAQIAS000000000, JAQIAR000000000, JAQIAQ000000000, JAQIAP000000000, JAQIAO000000000, JAQIAN000000000, JAQIAM000000000, JAQIAL000000000, JAQIAK000000000, JAQIAJ000000000, JAQIAI000000000, JAQIAH000000000, JAQIAG000000000, JAQIAF000000000, JAQIAE000000000, JAQIAD000000000, JAQIAC000000000, JAQIAB000000000, JAQIAA000000000, JAQHZZ000000000 and JAQHZY000000000.

## Supplementary Materials

The following supporting information can be downloaded at https://www.mdpi.com/article/10.3390/pathogens13090760/s1. Figure S1: Box-plot distribution of genome length from MSSP and MRSP strains isolated from canine infections. Statistical differences were calculated using Wilcoxon test, with significance indicated by * (*p* < 0.05); Table S1: Complete genomes of *Staphylococcus pseudintermedius* strains from canine and human hosts for comparative genomic and phylogenomic analyses; Table S2: Diversity of insertion sequences found in the 28 genomes of *Staphylococcus pseudintermedius* from this study, according to the ISFinder program; Table S3: Diversity of plasmids identified in the 28 genomes of *Staphylococcus pseudintermedius*, according to the PlasmidFinder program; Table S4: Comprehensive characterization of resistance and virulence gene profiles in 28 *Staphylococcus pseudintermedius* genomes.
